# A Nephrectomy—With Remarks

**Published:** 1903-07

**Authors:** J. G. Earnest

**Affiliations:** Atlanta, Ga.


### A NEPHRECTOMY—WITH REMARKS.*

By J. G. EARNEST, M.D.,
ATLANTA, GA.

The following case, referred to me by Dr. C. M. Paine, was first seen September 9, 1902. The patient, white, male, at thirty, stated that he had been in perfect health up to three weeks previous to that time, when he began to have pain in the neighborhood of the left kidney and noticed a flocculent white sediment in his urine. He also began about that time to have rigors followed by fever. I found him at ten o'clock in the morning with a temperature of 102, complaining of intense pain about the left kidney extending down into the front of the abdomen on that side. This pain was so great as to require the constant use of opiates. The physical examination showed a mass bulging out from the lower border of the ribs, reaching back to the kidney and extending downward to a point opposite the umbilicus. The mass was very sensitive. The quantity of fluid passed from the bladder was about normal and contained a large per cent. of white flocculent sediment which was found to be pus. A careful microscopic examination was made, especially looking for tubercle bacilli, but nothing of importance but pus was found. I suspected that there was probably a large stone in the kidney. It was finally settled that nothing short of surgical interference would give any relief. The patient was carried to the Halcyon Retreat September 22 and prepared for operation, which was done on the 23d of September. The usual lumbar incision was made, exposing the kidney as freely as the space between the lower rib and top of the ilium would ad-

* Read before Medical Association of Georgia, Columbus, April 17, 1903.

mit. The post-renal fat had been entirely absorbed. There was no pus outside the kidney. After pulling back the surrounding tissues it was found the kidney was entirely too large to be brought out of the incision. The organ was freely incised, when a quantity of pus, estimated by the gentlemen present to be not less than two pints, was discharged. There was no stone or evidence of tubercle or fungous growth found. The kidney was simply converted into a sack with walls about one third of an inch thick and perfectly smooth inside. The adhesions to the surrounding structures were so dense as to make a very difficult dissection. The vessels and ureter were tied off en masse with heavy silk and the wound moderately packed with five per cent. iodoform gauze. The external wound was partly closed—sufficient space being left for free drainage. This case has been reported in order that I might call attention to one feature of the case that seems to me extraordinary, that is the enormous quantity of pus passed for ten or twelve days before the operation. I noticed for several days what seemed a very unusual amount of pus in the urine, and so about eight or nine days previous to operating I began having all the urine he passed during the twenty-four hours voided into bottles of known capacity and set aside that I might estimate the quantity of pus. After standing twenty-four hours the pus was found to have settled down into a solid mass at the bottom. I am confident that there were not less than eight ounces of pus passed any day after I began a systematic estimate of the quantity. That was about eight or nine days. Whether this represented the pus-forming capacity of the sack or was partly the overflow of pus previously formed, I know not, but feel absolutely certain as to the quantity passed.

The patient made an uneventful recovery and four months after the operation was in perfect health, having gained about forty pounds.

---

A SURGEON should always pay much attention to the condition of a patient's nervous system. Some cases of neurasthenia present the most annoying train of symptoms after operations, in which the slightest point of annoyance is magnified into a matter of extreme importance.—*International Journal of Surgery*.

